# Evaluation of Injectable Hyaluronic Acid-Based Hydrogels for Endodontic Tissue Regeneration

**DOI:** 10.3390/ma14237325

**Published:** 2021-11-30

**Authors:** Esteban Astudillo-Ortiz, Pedro S. Babo, Rui L. Reis, Manuela E. Gomes

**Affiliations:** 1 3B’s Research Group, I3Bs—Research Institute on Biomaterials, Biodegradables and Biomimetics, University of Minho, Headquarters of the European Institute of Excellence on Tissue Engineering and Regenerative Medicine, AvePark, Parque de Ciência e Tecnologia, Zona Industrial da Gandra, Barco, 4805-017 Guimarães, Portugal; esteban.astudillo@ucuenca.edu.ec (E.A.-O.); pedrombabo@gmail.com (P.S.B.); rgreis@i3bs.uminho.pt (R.L.R.); 2 ICVS/3B’s—PT Government Associate Laboratory, 4805-017 Guimarães, Portugal; 3 Department of Endodontics, Area of Histology, School of Dentistry, University of Cuenca, Cuenca 010204, Ecuador

**Keywords:** hyaluronic acid, hydrogels, injectable, tissue engineering, endodontic tissue regeneration

## Abstract

Dental pulp tissue engineering (TE) endeavors to regenerate dentin/pulp complex by combining a suitable supporting matrix, stem cells, and biochemical stimuli. Such procedures foresee a matrix that can be easily introduced into the root canal system (RCS) and tightly adhere to dentin walls to assure the dentin surface’s proper colonization with progenitor cells capable of restoring the dentin/pulp complex. Herein was investigated an injectable self-setting hyaluronic acid-based (HA) hydrogel system, formed by aldehyde-modified (a-HA) with hydrazide-modified (ADH), enriched with platelet lysate (PL), for endodontic regeneration. The hydrogels’ working (wT) and setting (sT) times, the adhesion to the dentine walls, the hydrogel’s microstructure, and the delivery of human dental pulp cells (DPCs) were studied in vitro. Hydrogels incorporating PL showed a suitable wT and sT and a porous microstructure. The tensile tests showed that the breaking point occurs after 4.3106 ± 1.8677 mm deformation, while in the indentation test after 1.4056 ± 0.3065 mm deformation. Both breaking points occur in the hydrogel extension. The HA/PL hydrogels exhibited supportive properties and promoted cell migration toward dentin surfaces in vitro. Overall, these results support using PL-laden HA injectable hydrogels (HA/PL) as a biomaterial for DPCs encapsulation, thereby displaying great clinical potential towards endodontic regenerative therapies.

## 1. Introduction

About 13% of worldwide population has been subjected to some kind of endodontic treatment on at least one tooth [1,2]. However, the common root canal treatment turns the tooth non-vital. Given the absence of physiological homeostasis-maintenance mechanisms, non-vital teeth present a high risk of long-term failure [3,4]. After root canal treatment, the risk of tooth loss has been calculated to be around 10% after five years and close to 20% at ten years [5,6].

Consequently, during the last decades, therapies capable of maintaining tooth vitality, such as bleeding induction, or capable of regenerating endodontic tissues through tissue-engineered (TE) grafts have been proposed [7]. TE approaches are able to regenerate lost endodontic structures using biomaterials laden with biochemical cues and/or cells. Furthermore, they should enable the anastomosis with host tissues and promote the recruitment of suitable progenitor cells, allowing the simultaneous regeneration of the pulp and the dentin-pulp complex [8,9].

Hyaluronic acid (HA), or hyaluronan, is an important component of the extracellular matrix (ECM) in many tissues, including those derived from the neural crest, like the dental pulp [10]. HA has been explored for TE due to its biological and physicochemical features [11]; additionally, its clinical use has been approved by the US Food and Drug Administration (FDA) [12]. It is particularly interesting for TE applications because of its good biocompatibility, gel-forming properties, natural degradation, and ease of modification through its carboxyl and hydroxyl groups in order to produce stable hydrogels [13,14,15]. HA is also one of the major structural glycosaminoglycan components of the dental pulp extracellular matrix (ECM), and plays an essential role in several cellular tissue functions. The addition of hemoderivatives such as platelet lysate (PL) provides the hydrogel with an autologous source of growth factors and fibrin matrices necessary during the early stages of wound healing and tissue regeneration including pulp tissue [16]. In particular, the HA hydrogels incorporating platelet lysate (PL) have been shown to support the growth and differentiation of DPCs [11,17].

Therefore, herein we will evaluate an injectable self-setting hyaluronic acid-based hydrogel enriched with platelet lysate (HAPL) as a hydrogel for regeneration in endodontics. This hydrogel is formed by the spontaneous reaction between the aldehydes from aldehyde-functionalized hyaluronic acid (a-HA) and the hydrazides of adipic acid -hydrazide-modified HA (ADH-HA). The a-HA can further react with primary amines forming Schiff’s base bounds, e.g., with the amines of the dentin extracellular matrix (ECM) [18], which would assure a stable linking to the root canal walls fostering an effective cell colonization of the dentin.

## 2. Materials and Methods

### 2.1. Preparation of Hydrogels Precursors

The hydrogel precursors a-HA and ADH-HA were produced using standard protocols established by our group, as described elsewhere [11].

#### 2.1.1. Hydrazide-Modified Hyaluronic Acid (ADH-HA)

ADH-HA was produced by modifying the carboxylic acid moieties of the D-glucuronic acid with adipic acid dihydrazide. Adipic acid dihydrazide (ADH) (≥98%; Sigma-Aldrich, Saint Louis, MO, USA) was added to a 1 wt.% HA (Mw~1.5–1.8 MDa; Sigma-Aldrich, Saint Louis, MO, USA) solution prepared in Milli-Q water, at a molar ratio of 1 (ADH:HA). Then, the reaction occurred in the presence of N-(3-Dimethylaminopropyl)-N′-ethylcarbodiimide hydrochloride (≥98.0% Sigma-Aldrich, Saint Louis, MO, USA) (molar ratio 0.25; EDCI:HA) at pH 4.75, by addition of HCl dropwise, at room temperature, until no further pH variation was observed. The solution was then dialyzed firstly against diluted HCl containing 0.1 M NaCl (3 × 5 L, 48 h), followed by dialysis in diluted HCl (pH 3.5) (3 × 5 L, 24 h) and finally, against Milli-Q water. The resultant solution was frozen at −80 °C, recovered by freeze-drying, and stored until further use at −20 °C [11].

#### 2.1.2. Aldehyde-Modified HA (a-HA)

The a-HA was produced by sodium periodate (NaIO4) oxidation, as previously described [11]. In summary, HA (2.5 mmol; Sigma-Aldrich, Saint Louis, MO, USA) was dissolved in mili-Q water at 2% and an aqueous solution of sodium periodate (0.5 M, 5 mL) was added dropwise, stirring for two hours at room temperature in the dark. The reaction was stopped by adding ethylene glycol, and the mixture was transferred into a dialysis membrane (cut-off 14 MDa; Sigma-Aldrich, Saint Louis, MO, USA) and purified by dialysis against ultrapure water for three days. Finally, the dried a-HA product was obtained by freeze-drying and stored until further use at −20 °C and protected from light.

#### 2.1.3. Platelet Lysate (PL)

PL was prepared from human platelet concentrate by freeze/thaw cycles, using standard protocols, as previously described [19]. A set of 12 batches of platelet concentrate was subjected to three freeze/thaw cycles (−196 and 37 °C), aliquoted, and stored at −80 °C until needed. Just before use, vials were defrosted in a 37 °C water bath. Then, the cellular debris was removed by centrifugation at 4000× *g* for 5 min, and the supernatant was filtered through 0.45 µm filters inside a flow chamber.

#### 2.1.4. Preparation of PL-Laden HA Hydrogels (HAPL)

Hydrogel precursor solutions were prepared at room temperature by dissolving a-HA with PBS at 2% (*w*/*v*) (solution A) and ADH-HA in PL also at 2% (*w*/*v*) (solution B). As a control group, a solution of ADH-HA was mixed with PBS (solution C). After the hydrogel was well homogenized, the air bubbles were removed by short spinning the vials. Equal volumes of the precursor solution A and B were loaded in a double-barrel syringe and fitted with a static mixer placed at the outlet to produce the injectable HAPL, whereas equal volumes of A and C solution were loaded in the double-barrel syringe to produce the plain hydrogels (Control).

### 2.2. Characterization of Hydrogels

#### Working Time and Setting Time

**Figure 1:**
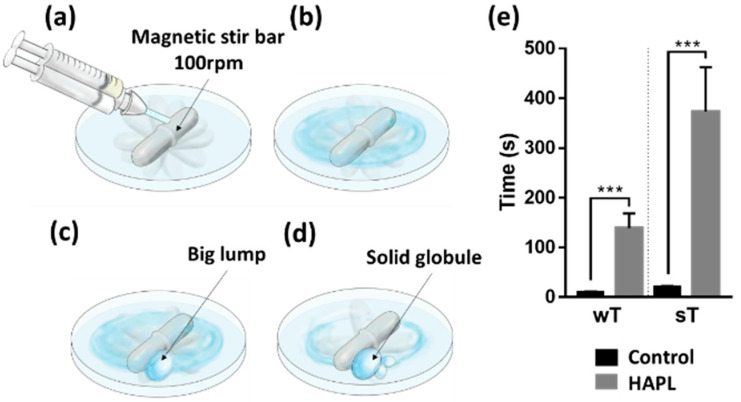
Working and setting time. (**a**) Injection of the hydrogel precursors directly above the magnetic stir bar. (**b**) As in the previous stage, the mixed solution keeps liquid flowing as the magnetic stir bar rotates. (**c**) Working time (wT) was registered when the first lump outline appeared, and the magnetic stir bar showed a slight impediment to turning. (**d**) Setting time (sT) was registered when the hydrogel formed a solid globule that prevented the normal rotation of the magnetic bar. (**e**) Graphic representation of the wT and sT obtained for the control and HAPL hydrogels. Values represent the mean ± SD of 5 tests. Symbols (***) denote study groups with statistically significant difference *p* < 0.0001.

The working and setting times of plain and PL-laden HA hydrogels were tested as previously described [11]. In brief, a 5 × 10 mm (diameter × length) magnetic stir bar was placed rotating at 100 rpm into the center of a glass petri dish at room temperature (Figure 1). Then, a 100 µL drop of ADH-HA solution in PBS or PL was injected over the magnetic stirrer. The crosslinking reaction was started by adding a 100 µL drop of a-HA solution. The working time was counted from the moment at which the precursors were mixed to the gel state, determined when a big lump starts to form from the mixed solutions. The setting time was determined when a solid globule that did not wet the Petri dish’s surface was formed. The assay was performed five times for each condition (Figure 1). The process was recorded using a 64 megapixels digital camera (Redmi note 8 pro, Xiaomi, Beijing, China) mounted at 10 cm from the samples. One previously trained observer was responsible for registering the data.

### 2.3. Preparation of Tooth Slice Samples

Tooth samples were prepared from human teeth, following an established procedure with some modifications [20,21]. Briefly, residual soft tissues were removed with a scalpel, and the dental surfaces were wiped down with 70% ethanol. Next, the teeth were transversally cut with a NTI Superflex diamond disc (Kerr, Orange, CA, USA), properly cooled to obtain slices of approximately 2-mm-thickness. The teeth slices were washed with PBS immediately after cutting.

To the mechanical tensile tests, 5 mm diameter dentin discs from the coronal region were polished using very fine grit sandpaper (3M, St. Paul, MN, USA) to obtain flat and smooth surfaces which were then cyanoacrylate bonded to the heads of 10 screws. The dentin discs’ opposite surface was also polished until they obtained a flat surface perpendicular to the large axis of the screw.

For the mechanical indentation assays and tooth slice organ cultures, the hole corresponding to the pulp chamber or root canal in the dentin slices of the cervical region was prepared, and standardized using a 1.2 mm high-speed straight cylinder diamond bur (Kerr, Orange, CA, USA), allowing a volume canal ranging between 29–43 mm^3^.

All the dentin samples were treated following the protocol reported for Aksel in 2020 with some modifications (Aksel et al., 2020). In summary, teeth slices were immersed in (1) 5% sodium hypochlorite for 2 min; (2) rinsed with 3 mL of milli-Q water; (3) EDTA 17% for 5 min; (4) rinsed with 3 mL of milli-Q water; (5) after that, the surfaces were conditioned with a-HA for 5 min; (6) finally, washed with PBS before injecting the hydrogels.

### 2.4. Assess the Adhesion of HAPL to the Dentine Walls

#### 2.4.1. Scanning Electron Microscopy (SEM) Analysis

HAPL’s interaction with the dentinal walls was analyzed under Scanning Electron Microscopy (SEM). HAPL hydrogels were injected into simulated root canals of dentin discs and set at 37 °C for 10 min. Then, the samples were stored at −20 °C for 24 h before being transferred to a −80 °C freezer for 48 h. Next, samples were submitted to a freeze-drying process for 48 h. Finally, each sample was mounted in an aluminum pin and the inner part of the hydrogel was exposed using a scalpel blade to cut the outermost layer, before coating with Au/Pd by sputtering for 40 s and with a 12 mA current. The samples were analyzed in a High-Resolution Field Emission Scanning Electron Microscope with Focused Ion Beam-Auriga Compact-Zeiss (Carl Zeiss Microscopy Gmbh, Oberkochen, Germany).

#### 2.4.2. Tensile Test

**Figure 2:**
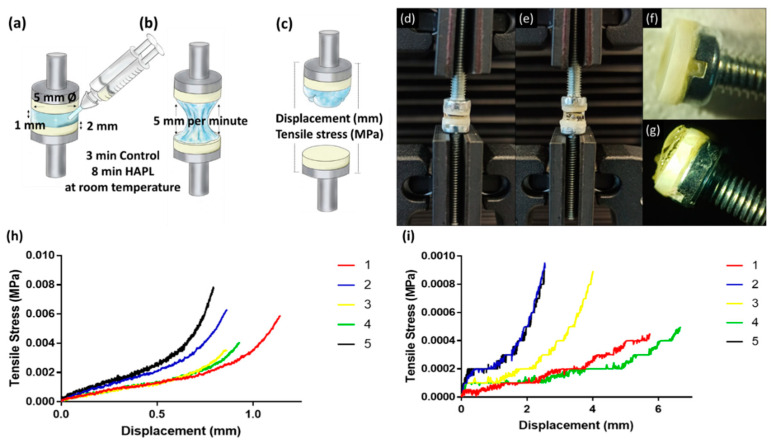
Tensile test. Schematic representation of the tensile test. (**a**) Loading of hydrogel in the 1 mm gap between the pre-conditioned dentin disks and zero-point position. (**b**) Dentin discs were continuously separated at a 5 mm per minute rate. (**c**) Breaking point values were registered after hydrogel loss of integrity or detachment from dentine. (**d**) Dentin disc glued to a screw head before the tensile test, and (**e**) sample prepared at the zero-point position, and (**f**) tensile test ongoing. (**g**) The hydrogel sample still adhered to the dentin disc after reaching the break point in the tensile test. (**h**) Tensile test graphic results of control hydrogel and (**i**) HAPL. Each line represents the tensile stress (MPa) curve of one individual sample.

**Figure 3:**
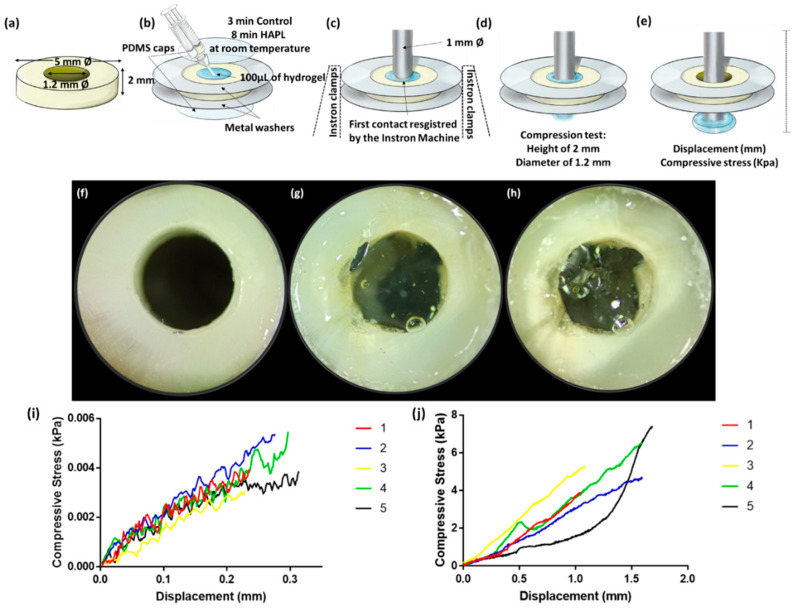
Indentation test. (**a**) Dentin disc obtained from a sound tooth. (**b**) Sample preparation. (**c**) Dentin disc mounted on the Instron machine before starting the test. (**d**) The metal stem is introduced at a continuous rate into the simulated canal filled with the hydrogel. (**e**) The breaking-point corresponding to hydrogel’s loss of integrity or detachment from the dentin walls. (**f**) Pre-conditioned dentin disk before injection of the hydrogel. (**g**) Simulated root canal filled with HAPL, ready to be used on the indentation test. (**h**) Same sample after the indentation test. (**i**) Indentation test graphic of control hydrogel or (**j**) HAPL. Each line represents the compressive stress (kPa) curve or one individual sample.

After completing the preconditioning step, the dentin discs surfaces were placed in direct contact, while on the opposite side, the screws were held with the clamps of the Instron machine (Instron, Buckinghamshire, UK). Using the Bluehill Universal software, the samples were separated 1 mm to inject the respective hydrogel using a double-barrel syringe until the surfaces were covered entirely (Figure 2). The distance between the samples was maintained until the setting time of the different solutions was completed. The control hydrogel was allowed to polymerize for 3 min and the HAPL for 8 min, both at room temperature. In the Bluehill software, the tension method was used and the irregular geometry field was configured, indicating a length of 5 mm and a rate of 5 mm per min (Figure 3). The assays started marking zero-point when the Instron machine registered the first value of tensile forces to standardize the measurements. Maximum load at break point was recorded for each specimen.

#### 2.4.3. Indentation Test

The dentin samples with the simulated root canal were glued with cyanoacrylate between two metal washers which allowed to stabilize the piece in the support clamps of the Instron machine. Before testing, all simulated root canals of the dentin samples were treated in pre-conditioned as above described. Once the pre-conditioning was complete, polydimethylsiloxane (PDMS) caps were placed in direct contact with the dentin surface of the bottom end of the simulated canal (Figure 3). Using the double-barrel syringe, approximately 100 µL of the corresponding hydrogel solution was injected into the simulated root canal, and a PDMS cap was placed in the top end of the simulated canal, and the hydrogel was let to set, to create smooth parallel surfaces. Then, the simulated root canals filled with the respective polymerized hydrogel were loaded immediately into the lower clamp of the Instron machine.

In the Instron machine, through the Bluehill Universal software, the compression test mode was used. A 1 mm diameter metal pin with a flat end was placed in the upper clamp in direct contact with the hydrogel with a trajectory directed towards the simulated root canal. Zero-point was registered when the Instron machine indicated the first contact between the metal stem and the hydrogel surface, and the last score corresponds to maximum load at break point for each specimen (Figure 3).

### 2.5. Evaluation of Hydrogel’s Ability to Deliver Stem Cells Endodontically

#### 2.5.1. Expansion of Human Dental Tissues Derived Cells

Human dental pulp cells (DPCs) were isolated from human third molars under the scope of previous studies [17]. Both of them were cultured in 75 cm^3^ TCP flasks with Dulbecco’s Modified Eagle’s Medium-low glucose (DMEM, Sigma- Aldrich, St. Louis, MO, USA) supplemented with 10% fetal bovine serum (FBS) (ThermoFisher Scientific, Waltham, MA, USA) and 1% antibiotic/antimycotic (ThermoFisher Scientific, Waltham, MA, USA). The culture medium was replaced twice a week and the cultures maintained semi-confluent. All cultures were incubated at 37 °C in a 5% CO_2_ high-humidity environment. Cells were used between passages 3 and 5 in this study.

#### 2.5.2. Encapsulation of DPCs

**Figure 4:**
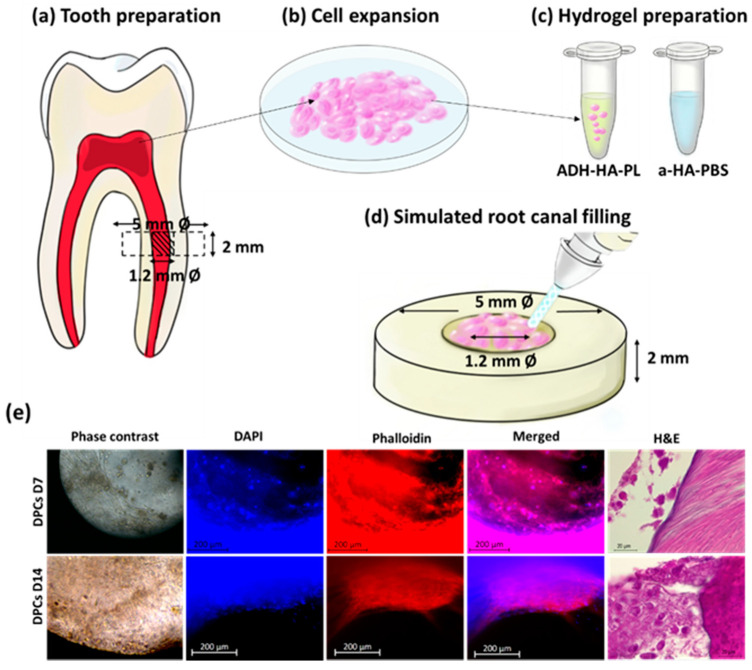
Hydrogels’ ability to deliver DPCs. (**a**) Schematic representative of the preparation of a tooth slice organ for cell culture. A human sound tooth was used to get DPCs. (**b**) DPCs cells isolated from the human sound tooth were cultured to test the hydrogel’s ability to deliver stem cells and (**c**) 8 × 106 of cells per mL were resuspended in ADH-HA-PL hydrogel precursor solution. (**d**) Using a double-barrel syringe, the hydrogels’ precursors were mixed and injected into preconditioned dentin discs of 5 mm diameter and 2 mm heigh with a hole standardized at 1.2 mm diameter corresponding to its root canal were used as tooth slice organ TSO. The TSOs were cultured to test the hydrogels’ ability to deliver dental stem cells. DPCs encapsulated in HAPL (**e**) TSO containing DPCs after 7 days and 14 days in culture by phase-contrast microscopy (10×), fluorescence microscopy (DAPI-Phalloidin staining; 20×) and representative histological section (H&E staining; 100×). Indentation test.

The preparation of the hydrogels was performed similarly to the procedure described in Section 2.1 but under sterile conditions. Briefly, a-HA was dissolved in DPBS, while ADH-HA was dissolved in PL, both at a concentration of 2 wt% overnight at room temperature. Then, the solutions were sterilized by UV irradiation (254 nm) for 30 min prior to cell encapsulation. DPCs cultures were trypsinized and resuspended in culture media, counted, and centrifuged at 300× *g* for 5 min, 22 °C. The supernatant medium was discarded and 8 × 106 cells per mL were resuspended in the respective ADH-HA/PL solution mixing up and down with a piston pipette. The cell mixture was added to barrel A of the double-barrel syringe, and barrel B was filled with a-HA (Figure 4).

#### 2.5.3. Tooth Slice Organ Cultures

After disinfected and pre-conditioned, simulated root canals 1.2 mm diameter and 2 mm of height the dentin slices were filled carefully with approximately 50 µL of the hydrogel laden with the corresponding cells and incubated at 37 °C for 30 min to form a solid gel (Figure 4). The tooth slice organs (TSOs) of each cell group were cultured in 24-well plates, 3 in basal media, and 3 in osteogenic media for 7 and 14 days (37 °C, 5% CO_2_), changing the culture media twice a week. The TSOs were evaluated during the whole assay by inverted microscopy. Subsequently, after culture time points, the TSOs were fixed in 10% formalin for 48 h and analyzed by fluorescence, histochemical, and immunohistochemical microscopy.

#### 2.5.4. Fluorescence Microscopy

After 7 and 14 days in culture, TSOs were washed carefully with PBS before and after fixation with 10% (*v/v*) neutral buffered formalin (ThermoFisher Scientific, Waltham, MA, USA) for 30 min at room temperature. After, the samples were incubated in a solution of 0.2% (*v/v*) Triton X-100 (Sigma-Aldrich, St. Louis, MO, USA) in PBS for 1 h at room temperature with slight movements. Then, samples were incubated in 1 mL of the PBS solution containing 4,6-diamidino-2-phenyindole dilactate (DAPI, Biotium, San Francisco, CA, USA) 1:10,000 *v/v* Phalloidin (Phalloidin–Tetramethylrhodamine B isothiocyanate from Amanita phalloides Sigma-Aldrich, St. Louis, MO, USA) 1:200 *v/v*, for 1 h at room temperature, under mild agitation. Subsequently, the samples were washed 3 times in PBS for five min to reduce the background fluorescence. Then, the samples were visualized under a Fluorescence Inverted Microscope (Zeiss, Axio Observer Z1, Göttingen, Germany), and representative micrographs were taken.

#### 2.5.5. Histological Processing

After fluorescence analysis, the TSOs were fixed again by 48 h and demineralized using a decalcifying agent composed of 22.5% formic acid and 10% (*w*/*v*) sodium citrate for 2 weeks. The demineralization was confirmed chemically, using a standard protocol [22]. The test was performed daily, mixing 0.5 mL of the decalcification solution supernatant with 1.0 mL of citrate-phosphate buffer, and 2.5 mL of saturated ammonium oxalate solution into a falcon tube. After 20 min, a calcium precipitate is formed if the decalcification is still occurring. If positive the tooth samples were washed with distilled water and immersed in fresh decalcifying solution. Otherwise, the test was repeated the following day without refreshing the decalcifying solution. The end point was considered when the test screened negative results for two consecutive days.

Then, the samples were processed and embedded in paraffin wax for histological and immunohistochemical examination. Sections were cut at 4 µm and routinely stained with hematoxylin and eosin.

### 2.6. Statistical Analysis

Data were processed using Microsoft Office Excel 2019 and analyzed using GraphPad PRISM 7 (GraphPad Software Inc., La Jolla, CA, USA). Friedman’s test was used to detect differences across the measures, followed by an unpaired t-test with Welch’s correction to find differences between the group samples. Results were presented as mean ± standard error of the mean. Statistical significance and associated degree of confidence (*p* < 0.05) are represented by symbols stated in the graphs.

## 3. Results

### 3.1. Working Time (wT) and Setting Time (sT)

The measurements of the apparent in situ gelation time of both hydrogel formulations are summarized in Table 1, described as working and setting times. The control hydrogel started its polymerization process at 10 s (±1.4 s) after mixing the precursors and finished after 20 s (±1.6 s) (Table 1). The incorporation of PL significantly increased the wT to 2 min and 18 (±29), and the sT increased to 6 min and 13 s (±89 s) (*p* = 0.0001) (Figure 1).

### 3.2. Dentin Pre-Conditioning and Hydrogel Microstructure

**Figure 5:**
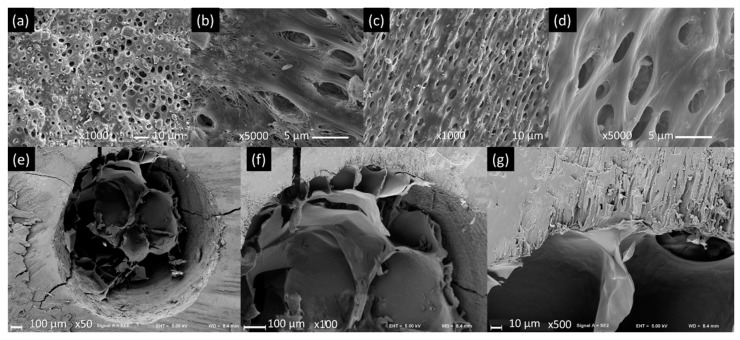
Hydrogel microstructure and interaction with dentin. Representative (**a**) dentin of the simulated root canals before the pre-conditioning observed under SEM at 1000× and (**b**) 5000×. (**c**) The same simulated root canal after pre-conditioning 1000× and (**d**) 5000×. (**e**) Simulated root canal filled with HAPL observed under SEM at 50×, (**f**) 100×, and (**g**) 500×. This is a figure. Schemes follow the same formatting.

Before pre-conditioning, it was possible to observe that the collagen fibers were exposed and the dentin surfaces covered by debris and smear layer (Figure 5a,b). After pre-conditioning, less debris was present while the collagen fibers were dipped by the a-Ha layer (Figure 5c,d). The HAPL hydrogels presented a heterogeneous, interconnected porous structure, in which larger pores could be observed in the central part, surrounded by smaller pores in the periphery (Figure 5e). When analyzing the hydrogel-dentin interface through SEM, using a magnification of 100×, it was possible to see a direct contact between both (Figure 5f). Analyzing at higher magnifications (500×) made visible a continuum between hydrogel and dentin matrix, and the hydrogel penetration into the dentin tubules present in the walls of the simulated root canal (Figure 5g).

### 3.3. Ability of the Hydrogels to Adhere to the Dentine Walls

After the tensile test, no hydrogel sample was detached from the dentin surfaces. The breaking point was always located in the extension of the hydrogel (Figure 3g). The control hydrogel withstood a displacement of 0.9 ± 0.1 mm and tensile stress of 5.6 ± 2.0 kPa before the break point (Figure 3h; Table 2). In contrast, the HAPL resisted a 4.3 ± 1.9 mm displacement and tensile stress of 0.7 ± 0.2 kPa (Figure 3i; Table 2).

Likewise, no hydrogel samples were detached from the dentin after the indentation test. In all the assays, the metal stem hole through the hydrogel without compromising the attachment to the dentin (Figure 3h). Before breakpoint, the control hydrogel supported a displacement of 0.269 ± 0.038 mm and compressive stress of 0.004 ± 0.001 kPA (Figure 3i; Table 2). While, after adding PL to the hydrogel, its resistance to displacement was 1.336 ± 0.306 mm and supported compressive stress of 5.096 ± 1.405 kPa before the break point (Figure 3j; Table 2).

### 3.4. Hydrogel’s Ability to Deliver Dental Derived Cells

One of the most important hydrogel characteristics for endodontic TE is the ability to deliver stem cells, support their proliferation and colonization of dentin surface, aiming the formation of a dentin/pulp complex-like tissue.

In this assay, HAPL remained stable inside the tooth slice organ (TSO), however it presented signs of degradation when removed from wells to be processed. For extended culturing times (day 14), the hydrogel almost entirely degraded and was replaced by dental pulp-like tissues that were attached to the walls of the simulated root canal.

The presence of cells after each timepoint was confirmed through DAPI-phalloidin staining using fluorescence microscopy. The immunofluorescence analysis showed that the DPCs presented a typical spindle-like shape during the first seven days and allowed migration of DPCs towards the dentin walls (Figure 4e). From day ten onwards, the hydrogel was degraded centrifugally, and it was possible to observe that the DPCs gradually converged towards the dentin walls, forming conglomerates.

Histologically, after 7 days, it was possible to observe cells approaching the dentin surface, depositing an incipient ECM. On day 14, the histological analysis showed the deposition of a loose-type extracellular matrix composed of few fibers and abundant ground substance derived from DPCs near dentinal walls.

## 4. Discussion

This work aimed to evaluate whether the injectable aldehyde-hydrazide hyaluronic acid-based hydrogel enriched with platelet lysate encapsulating DPCs has the suitable properties and abilities for its potential use in regenerative endodontics. The analysis performed in this in vitro study allowed us to evaluate the gelation characteristics, injectability, bound to dentin walls, and cell-supportive properties of a class of injectable self-setting aldehyde-hydrazide hyaluronic acid-based hydrogel enriched with platelet lysate as a TE construct aiming endodontic tissue regeneration.

The choice of an injectable hydrogel for endodontic regeneration is based on its similarities to soft tissues, providing an environment physiologically suitable for cell growth. The hydrogels mimic the ECM properties such as high-water content, controllable porosity, and mechanical and physicochemical properties, supporting the natural functions of cells [23]. Specifically, in the case of tunable aldehyde-hydrazide-based hydrogels it is also possible to allow the development of cell morphologies similar to the original tissues [23]. Moreover, the reaction between aldehydes and hydrazides forms Schiff bases capable of generating linkages with the collagen fibers of the dentin [23], enhancing the integration of the hydrogel TE graft into host’s tissues.

Although the expected therapeutic function of this TE approach is not the same as an endodontic sealer, i.e., its function (aim) is not to seal the root canal but to regenerate the endodontic tissues, we will discuss some characteristics against endodontic sealers due to the similarities in its handling and application. The injectability of these hydrogels is one of the main advantages for endodontic applications, allowing the material to fill root canal systems in all their complexity, emulating a technique often applied in silicon-based sealers [24]. As herein observed, the hydrogel tested can be easily applied into simulated root canals prepared using standard instrumentation and preconditioning protocols. Thus, it is fair to assume that it will find familiarity among clinicians. At the same time, its self-setting properties assure that polymerization occurred in the whole injected solution, maintaining the same properties along the entire root canal.

The present study showed an increase in the setting and working time after adding PL to the HA hydrogel solutions. The gelation of the hydrogels herein studied is achieved by Schiff reaction. The Schiff reaction is a chemical reaction involving a dynamic covalent imine bond formation via the crosslinking of amine groups and aldehyde groups. The reaction kinetics depend on the pH, temperature, and type of aldehydes/amines involved in the reaction [25]. The aldehyde reactant, the oxidized HA, is the same for both the formulations. However, in the HA hydrogels the reaction occurs only with the hydrazides of the ADH-HA, while in the HAPL, the Schiff reaction can also occur with the primary amines of the PL proteins. Given that at physiologic pH the Schiff reaction with primary amines is slower and less stable than the hydrazone bound formation [23,25], the gelation time, and consequently the wT and sT, is increased. Moreover, the sodium citrate used as an anticoagulant in the platelet concentrate unit used to prepare PL can also interfere with the amine-aldehyde cross-linking [11,19]. Regarding the working time, the HAPL is faster (2.18 ± 0.29 min) than the referred values of established endodontic sealants, such as those studied by Zhou in 2013, including resin-based endodontic sealers such as AH plus (240 ± 40 m) and silicone-based sealers such as Guttaflow (15 ± 5 min) [24]. However, it is necessary to clarify that the methods used to measure the working time were different. The increase of the working time promoted by incorporating PL could be enough for a future clinical application. So, the consequences of a short working time are related to the lack of flow inside the root canal and the little or no capacity to reach its total length and its anfractuosities [26]. The working time of HAPL is comparable to that of Dycal (2.20 min), a material that was initially designed as an endodontic sealer [27] but was later used as a pulp protector due to its reaction speed. However, Dycal develops its maximum physical properties almost immediately (sT 2.5 to 3.5 min), unlike HAPL (6.13 min) [28]. Moreover, the swelling characteristic in hydrogels [29] would refill any slight filling deficiency in the canal, complementing it with sonic or ultrasonic activation to enhance fluency and adaptation [30].

The setting time of the HAPL hydrogel (6.13 ± 1.29 min) is lower than endodontic sealers as AH plus (690 ± 90 min) and Guttaflow (42 ± 6 min). However, for a future clinical application, a working time of 6 min would allow a comfortable application during a clinical session. Moreover, taking into consideration that the canal must be sealed with a bioceramic material to close the top end of the cavity, then with glass ionomer and a composite resin as was used in recent clinical case reports [31,32], time management is critical for the comfort for the patient.

Concerning the microstructure, the hydrogel presented open pores and interconnections that would facilitate the migration, nutrition, and proliferation of DPCs inside [33,34]. These results align with previous studies reported by other authors [11,17]. Furthermore, the microstructural analysis of the hydrogel also allowed us to observe its penetration into the dentin tubules and its close interaction with the dentin matrix collagen fibers. The pre-conditioning of the dentin surface, as herein performed, allows the tight interaction of the hydrogel with dentin matrix both by (1) transforming the reactive amines of dentin collagen into HA with reactive aldehydes by Schiff’s base reaction, thus bridging the reaction of the hydrogel matrix with the protein matrix of the dentin [23,35], and (2) creating a moist and homogeneous surface, facilitating the penetration of the hydrogel into the microtubules. This fact provides the hydrogel physical micro-adhesions that reinforce its bond to the dentin surface [18,36].

The tensile and indentation tests confirmed the above described. The hydrogel samples always remained attached to the dentin in both cases, while the breaking point was located within the hydrogel structure. Therefore, the data obtained explain the resistance values on themselves and assume that the values corresponding to the hydrogel-dentin interface are superior to those obtained. Moreover, the present in vitro study demonstrated that the incorporation of PL increased the resistance to the displacement from 0.9 ± 0.1 to 4.3 ± 1.9 mm at the same time that decreased the resistance to tensile stress from 6 ± 2 to 0.7 ± 0.2 kPa in the tensile test using an Instron Machine. In other words, PL incorporation into the HAPL hydrogel raises its flexibility while reducing its resistance to tensile forces, similar to the reported in the existing literature [11,17,37].

In the present study, HAPL was shown to support the delivery of cells to simulated root canals. HA hydrogels have been related to decreased cell proliferation and cytotoxicity [10,12]. PL-laden HA hydrogels demonstrated a positive effect on DPCs viability and proliferation, as was reported in other studies performed by our research group [11,17,37]. The release of PDGF and other mitogenic growth factors, and the presence of fibrin could explain the better performance of the PL-laden HA hydrogels: PDGF stimulates the proliferation of mesenchymal cells, and fibrin provides extra docking sites for cells to adhere and proliferate [17].

Over fourteen days, the hydrogels fostered the migration towards the dentin walls in vitro. Using phase-contrast microscopy, the cells encapsulated in the hydrogel migrated towards the periphery of the root canal while degrading the hydrogel and forming their matrix (Figure 4f). After H&E staining, it was possible to confirm the presence of DPCs, which showed signs of migration towards the dentin walls as soon as 1 week after implantation (Figure 4g).

Once there, dentin matrix would encourage DPCs adhesion, proliferation, and differentiation into odontoblast-like cells due to the presence of specific growth factors in its demineralized matrix, as was reported by Huang, Liu, and Salehi [38,39,40]. However, further investigation must be performed to confirm the latter in three-dimensional culture conditions or an in vivo assay because, in static culture, the nutrients and oxygen cannot flow efficiently throughout the three-dimensional matrices, which causes cell necrosis in the center part of the construct, as was reported by Tayebi [41].

As previously reported, HA hydrogel formulations similar to the HAPL herein studied degrade quite fast, particularly in the presence of hyaluronidase, an enzyme expressed in inflamed or remodeling tissues [1]. In general, Schiff bases are characterized by poor degradation, but aldehyde-hydrazide-based hydrogels form a subclass of Schiff’s base called hydrazone bond which presents excellent ability of hydrolysis in aqueous environments [18]. Therefore, to enhance the stability of the material against degradation, some researchers have reinforced the hydrogel with materials such as graphene or cellulose, obtaining satisfactory results in vitro [11,19,42,43]. However, we prefer not to use these reinforcements because it is still unknown whether they could generate foreign body reactions at the periapical zone as the classical cases reported by Koppang and Nair [44,45]. Moreover, HAPL degradation was centrifugal in all the samples, which means it began at the level of the pulp core and spread towards the periphery along the time. PL-laden HA hydrogels have been shown to entrap angiogenic, chemotactic, and morphogenic factors, such as vascular endothelial growth factor (VEGF) and platelet-derived growth factor (PDGF), among others, which are released in a hydrogel degradation-dependent manner [11,16,17]. This centrifugal degradation triggers the release of chemotactic growth factors that could allow a host tissue invasion carrying blood vessels in the core of the newly formed tissue. This fact gives an option to overcome the issues related to the regeneration of the complex hierarchical distribution present on the dental pulp tissues, i.e., a vascularized and innervated pulp core composed of loose connective tissue surrounded by specific dental cells such as odontoblasts [14].

## 5. Conclusions

The HAPL injectable and self-setting hydrogel provides familiar and straightforward applicability in endodontics. PL incorporation to the aldehyde-hydrazide hyaluronic acid-based hydrogel increases working time and setting time to handier levels allowing a suitable root canal filling. Furthermore, the hydrogel adheres tightly to the dentin matrix and penetrates the dentin tubules. Together with hydrogels resilience to displacement, this material is suitable for the function and the forces it will be subjected. Envisioning TE approaches aiming endodontic regeneration, the hydrogel is suited to deliver DPCs into instrumented and conditioned root canals, favored cell migration towards the dentin walls, and after the hydrogel degradation, it was replaced by extracellular matrix derived from DPCs. In a nutshell, HAPL hydrogel is a promissory biomaterial that demonstrates excellent performance needs further investigation for its potential clinical application in regenerative endodontics.

## Figures and Tables

**Table 1:** Descriptive statistics. Time in seconds (s) corresponding to the hydrogels’ working and setting time.

Material	Test	N	Mean (s)	SD (s)	Min (s)	Max (s)
*Control*	wT	5	10.0	1.4	8	12
*HAPL*	wT	5	138.8	29.6	105	178
*Control*	sT	5	20.8	1.6	19	23
*HAPL*	sT	5	373.4	89.1	302	516

**Table 2:** Descriptive statistics. Tensile and indentation tests.

Test	Material	Measure	1	2	3	4	5	Mean	SD
Tensile	Control	Displacement (mm)	1.1412	0.8674	0.8643	0.9301	0.7943	0.9194	0.1329
		Tensile stress (MPa)	0.0061	0.0061	0.0043	0.0043	0.0082	0.0058	0.0016
	HAPL	Displacement (mm)	5.7341	2.5421	4.0172	6.6927	2.5673	4.3106	1.8677
		Tensile stress (MPa)	0.0004	0.0009	0.0009	0.0005	0.0009	0.0007	0.0002
Indentation	Control	Displacement (mm)	0.2333	0.2815	0.2266	0.2906	0.3142	0.2692	0.0379
		Compressive stress (kPa)	0.0040	0.0054	0.0031	0.0055	0.0038	0.0043	0.0010
	HAPL	Displacement (mm)	1.0541	1.6003	1.0902	1.6001	1.6834	1.4056	0.3065
		Compressive stress (kPa)	3.9074	4.6874	5.2862	6.5021	7.4143	5.5594	1.4047

## Data Availability

The data used in this study can be accessed from the authors upon reasonable request.
